# Atrial Septal Aneurysm Leading to Ischemic Stroke: A Case Report and Literature Review

**DOI:** 10.7759/cureus.60955

**Published:** 2024-05-23

**Authors:** Shamaiza Waqas, Sean Dawes, Jorawar Brar, Waqas Abid, Amaraja Kanitkar

**Affiliations:** 1 Internal Medicine, Ascension Macomb-Oakland Hospital, Warren, USA; 2 Critical Care Medicine, Ascension Macomb-Oakland Hospital, Warren, USA; 3 Cardiology, Ascension Macomb-Oakland Hospital, Warren, USA; 4 Radiology, Ascension St. John Hospital, Detroit, USA; 5 Intensive Care Unit and Pulmonology, Ascension Macomb-Oakland Hospital, Warren, USA

**Keywords:** literature review, case report, ischemic stroke, atrial septal defects, atrial septal aneurysm

## Abstract

Stroke is the most common cause of death and disability in the world, and ischemic etiology plays a major role. Atrial septal aneurysm (ASA) is a localized saccular deformity of the atrial septum, associated with ischemic stroke independently or in association with other atrial septal defects (ASD). There is a higher incidence of stroke in the population with ASD. In these patients, the presence of ASA is an important predictor of recurrent stroke. This is a case of ischemic stroke in a 44-year-old who presented with sudden-onset right-sided body weakness, expressive aphasia, and non-specific confusion for one hour, with an initial National Institutes of Health Stroke Scale (NIHSS) score of 7. CT angiogram revealed occlusion of the M3 branch of the left middle cerebral artery in the left lateral frontal lobe. Code stroke was called, and the patient was given tenecteplase (TNK), after which her right-side weakness and aphasia resolved. Trans-thoracic echo with bubble study showed ASA with positive bubble study. Lone ASA or ASA with concomitant ASD poses a higher risk of recurrent stroke in younger patients, especially those without significant risk factors for strokes. Patients with ASA and concomitant ASD are at high risk for recurrent ischemic stroke and should be kept under surveillance with continued medical therapy. We present a case of ischemic stroke caused by ASA and a review of the current literature and case reports documenting cases with similar presentations.

## Introduction

Stroke is the most common cause of death and disability in the world, and 13.7 million new cases of stroke were noted in 2016, out of which around 87% were secondary to ischemic strokes [1].

Atrial septal aneurysm (ASA) is a localized saccular deformity of the atrial septum that bulges into the right or left atrium. The incidence of stroke in the population with ASD is around 10% [2]. In these patients, the presence of ASA is an important predictor of recurrent stroke [3]. It can be diagnosed with transthoracic echocardiography (TTE) or with transesophageal echocardiography (TEE). The frequency of the anomaly in the general adult population is low (2.2%) [4]. A pooled analysis of data from two prospective observational studies and randomized trials analyzing the effect of shunt size vs the presence of ASA in patients with patent foramen ovale (PFO)-associated stroke showed that the presence of ASA is a stronger predictor for recurrent ischemic stroke than shunt size [3].

In the literature, it has been reported to be associated with ischemic stroke independently or in association with other atrial septal defects (ASDs) [4,5]. A prospective study done by Cabanes et al. showed that ASA and PFO have synergistically increased the association with cryptogenic stroke [5].

We present a case of ischemic stroke caused by ASA, along with the analysis of existing literature and case studies detailing instances with analogous clinical presentations.

## Case presentation

This is a case of a 44-year-old woman, with a past medical history of hypertension for five years, migraine since childhood, and seizures for the last nine months. The patient was on topiramate for seizures, sumatriptan for migraine, and lisinopril, nifedipine, and metoprolol for hypertension. The patient presented to the emergency department with sudden-onset right-sided body weakness, expressive aphasia, and non-specific confusion that started one hour prior to presentation.

Vitals on presentation revealed a blood pressure of 147/86 mmHg, heart rate of 93 bpm, respiratory rate of 18 breaths/min, and oxygen saturation of 99% on room air. A neurological exam was significant for expressive aphasia, with 1/5 hemiparesis of the right arm and right leg and no purposeful movement. There were no sensory deficits. The patient was alert and oriented to time, place, and person. The initial National Institutes of Health Stroke Scale (NIHSS) score was 7.

**Figure 1:**
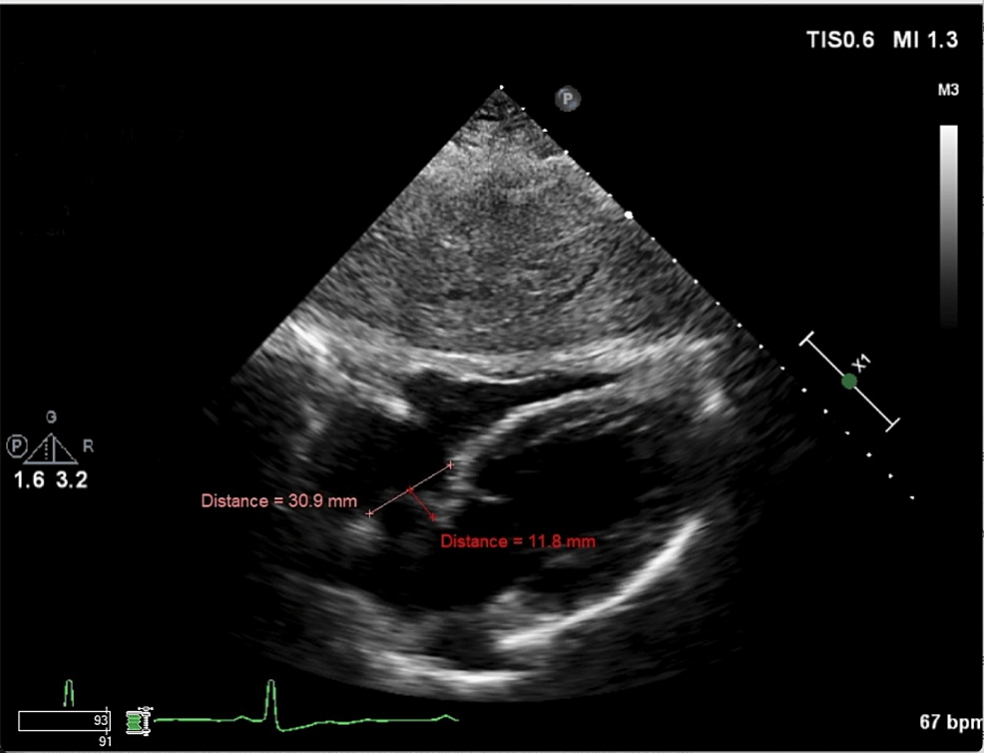
TTE in the subcostal view illustrating the oscillation of the atrial septal aneurysm during diastole. The aneurysm oscillates 11.8 mm towards the left atrium during ventricular diastole TTE: transthoracic echocardiography

**Figure 2:**
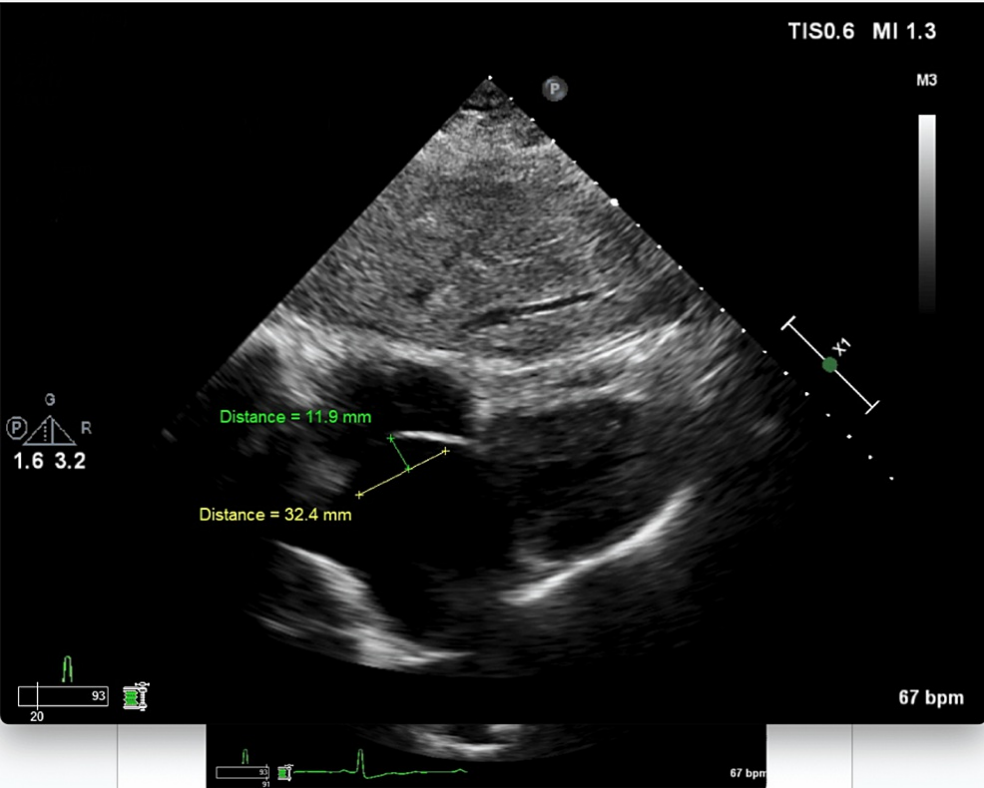
TTE in the subcostal view illustrating the oscillation of the atrial septal aneurysm during systole in the subcostal view. The aneurysm oscillates 11.9 mm towards the right atrium during ventricular systole TTE: transthoracic echocardiography

**Figure 3:**
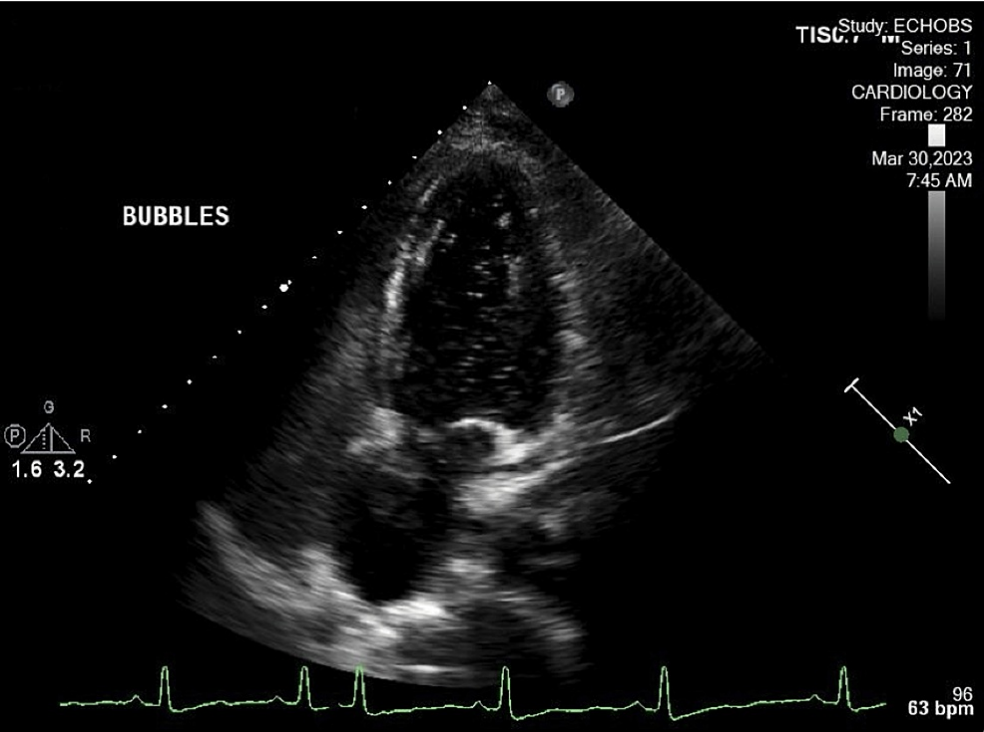
TTE in a four-chamber view illustrating the positive agitated saline bubble study. Agitated saline is seen clearly within the left atrium and left ventricle TTE: transthoracic echocardiography

**Figure 4:**
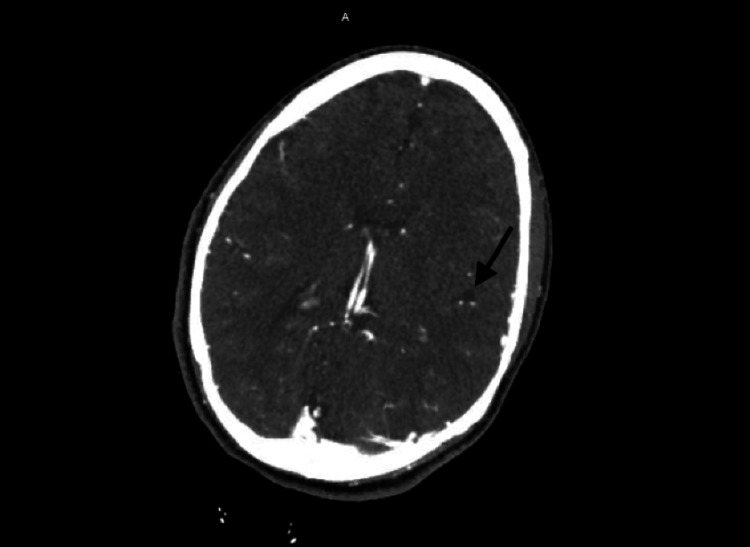
CT angiogram of brain showing occlusion of the M3 branch of the left middle cerebral artery in the left lateral frontal lobe

There was a focal moderate stenosis at the ostium of both vertebral arteries. Tortuosity of intracranial and neck vessels could be seen in the setting of long-standing hypertension. The stroke workup included TTE with bubble study. Figures 1-2 show the subcostal view of TTE, illustrating the oscillation of the ASA during diastole and systole, respectively. TTE was significant for thickened atrial septum, with a positive agitated saline study (Figure 3). CT angiogram revealed occlusion of the M3 branch of the left middle cerebral artery in the left lateral frontal lobe (Figure 4). The cardiologist read the report as a redundancy of the septum, with borderline criteria for ASA. The ASA was considered borderline based on the ASA definition of 15 mm excursion cutoff. The urine drug screen was positive for cannabis only. A thrombophilia panel was not done during the hospital stay as the patient's age was more than 40 years and she did not have any previous thrombotic event or miscarriage history; this is one of the limitations of this case report.

Clinical course

A code stroke was called in the emergency department, with an initial NIHSS score of 7. After ruling out intracranial hemorrhage and ruling out additional contraindications for thrombolysis, tenecteplase (TNK) 0.25 mg/kg was given to the patient. The NIHSS score improved to 5 subsequently after TNK. Neurology and neuroendovascular surgery were consulted. No acute intervention was planned by neuroendovascular surgery as the patient’s NIHSS score continued to improve, and the patient was transferred to the ICU for close monitoring. The patient tolerated the TNK well, and after six hours, her NIHSS score improved to a score of 1. Prior to discharge, the patient’s right arm hemiparesis, right leg hemiparesis, and expressive aphasia resolved completely. Cardiology was consulted for positive agitated saline and concern for ASA, as described on the TTE. Cardiology advised outpatient TEE for further characterization of the ASA. Prior to discharge, the patient was started on Plavix (clopidogrel) and statin by the neurologist for concern of atherosclerotic disease. She was discharged home and was advised to follow up in the clinic for further management.

## Discussion

ASA is primarily a congenital malformation of the interatrial septum, but it can also develop secondary to pressure differences between the left and right atrium [4,6]. It is a saccular deformity that can bulge into either side of the atrium. In the literature, different criteria are used to define ASA. Silver et al. [7] used more than 10 mm protrusion of atrial septum into either light or left atrium. Hanley et al. [8] defined ASA as at least a 15 mm protrusion of the atrial septum. In a large series of studies diagnosing ASA on TEE, Pearson et al. used the criteria of more than 10 mm excursion. Several other studies used definitions of 10 mm outpouching of the mitral portion of the septum as criteria [9]. An established guideline or cut off reference point is lacking for ASA in the literature. In the above case, the patient had a protrusion of more than 10 mm, which meets the definition of ASA based on several prior studies [7,9-11]. ASA can present alone without any other cardiac abnormality, or it can be associated with other cardiac atrial septal defects (ASDs) [5]. Type II ASD, patent foramen ovale (PFO) [5,7,8], and mitral valve prolapse (MVP) are commonly associated with ASA [5,9]. In a study done by Mügge et al., ASA was associated with type II ASD in 19% of patients [12].

ASA is associated with thromboembolic phenomenon and ischemic stroke independently [5,12] and in association with other septal defects such as PFO [5] (Table 1). It is proposed that the association of ASA and PFO has a marked synergistic effect as a causal factor for cryptogenic ischemic stroke. A study evaluating the presence of ASA and PFO as risk factors in younger patients showed that they are both associated with stroke independently and have a synergistic effect [5] (Table 1). ASAs of > 10 mm excursion are also associated with an eight times higher risk of stroke as compared to ASA of < 10 mm [5]. The mechanism by which ASA is associated with an increased risk of stroke is uncertain. However, two phenomena are speculated [6]. The first one is that a pseudo-cavity formation by ASA and associated increased local blood stasis can lead to thrombus formation [4,6]. The second one is an associated concomitant PFO [6]. Treatment for stroke associated with ASA alone or with PFO is medical therapy and/or surgical closure. Studies have shown that patients who have ASA associated with PFO are at an increased risk for recurrent stroke. These subgroups of patients are considered high-risk PFOs [3]. The Randomized Evaluation of Recurrent Stroke Comparing PFO Closure to Established Current Standard of Care Treatment (RESPECT) trial and aggregate data meta-analyses of all randomized controlled trials have shown that stroke patients with high-risk PFOs are a sub-group of patients that will likely benefit from PFO closure rather than medical therapy alone. The risk of stroke as a sequelae of PFO closure also decreased significantly after closure [2]. Further prospective studies are needed in this area. It has been postulated that ASA can be associated with atrial arrhythmias [8]. This area, however, needs further studies. ASA can be diagnosed with transthoracic echocardiography and/or transesophageal echocardiography. In diagnosing ASA, literature has shown that transesophageal echocardiography is superior to transthoracic echocardiography [10,11]. In our case, the ASA with interatrial defect was seen clearly on the TTE with bubble study (Figures 1-3).

**Table 1:** Literature review showing atrial septal aneurysm as a risk factor for cryptogenic stroke, lone or with concomitant atrial septal defects

Authors	Methods	Age	Risk of recurrent stroke with ASA	ASA size Vs associated ASD	Conclusion
Turc et al. 2020 [3]	Pooled individual patient data from 2 prospective observational studies and the medical arms of 2 randomized trials	Of 898 patients (mean age 45.3 years)	Over a median follow-up of 3.8 years, 47 (5.2%) patients experienced a recurrent stroke.	178 (19.8%) had ASA with large PFO, 71 (7.9%) ASA with nonlarge PFO, 397 (44.2%) large PFO without ASA, and 252 (28.1%) nonlarge PFO without ASA.	In patients with PFO-associated stroke, ASA is a more important predictor of recurrent stroke than shunt size These results can help to better identify those patients with a high risk of stroke recurrence under medical therapy who may derive the most benefit from PFO closure.
Razaq et al. 2012 [4]	Case report	6 years	__	Small ASA, no associated ASD.	An isolated small ASA may be a possible risk factor for ischemic stroke even in the pediatric age group.
Mügge et al. 1995 [12]	Retrospective study	195 patients 54.6±16.0 years	__	ASA was defined as a protrusion of the aneurysm >10 mm. ASA was an isolated structural defect in 62 patients (32%). In 106 patients (54%), ASA was associated with interatrial shunting (atrial septal defect, n=38; patent foramen ovale, n=65; sinus venosus defect, n=3). In only 2 patients (1%), thrombi attached to the region of the ASA were noted.	The most common abnormalities associated with ASA are interatrial shunts, in particular patent foramen ovale. In this retrospective study, patients with ASA (especially with shunts) showed a high frequency of previous clinical events compatible with cardiogenic embolism; in a significant subgroup of patients, ASA appears to be the only source of embolism,
Wong et al. 2014 [13]	Case report	49 years	Bi-hemispheric ischemic strokes, with the two strokes occurring over the course of ten days.	Small Left atrial septal pouch, no associated ASD.	Left atrial septal pouch was a likely cause of stroke.

## Conclusions

Lone ASA or ASA with concomitant ASD poses a higher risk of recurrent stroke in younger patients, especially those without significant risk factors for stroke. In patients with cryptogenic stroke, ASA and other ASDs should be ruled out as a cause of stroke to avoid recurrent stroke in the future. Patients with ASA and concomitant ASD are at high risk for recurrent ischemic stroke and should be kept under surveillance with continued medical therapy.
